# Nutrition, Cell Signalling, Mitochondrial Function, and Chronic Non-Communicable Disease

**DOI:** 10.3390/ijms27073303

**Published:** 2026-04-05

**Authors:** Russell Phillips

**Affiliations:** McKenzie Clinic, Sunshine Coast 4556, Australia; rustyp133@gmail.com

**Keywords:** mitochondria, extracellular vesicles, redox signalling, insulin signalling, nutrient sensors, AMPK, mTOR, autophagy, apoptosis, nutrition

## Abstract

Cellular homeostasis is a dynamic process which balances anabolic processes with catabolic and recycling processes. These processes require nutrients, which are converted to energy to fuel the complex interactions of intracellular signalling. Cellular health requires that, on average, energy input and energy requirements are matched. Cells contain a nutrient-sensing mechanism which controls the balance between anabolism and catabolism. Normal intracellular functions generate products which regulate signalling pathways, and health at a cellular level requires a fluctuation between relative nutrient abundance and relative nutrient scarcity. This allows clearance of damaged intracellular molecules and organelles. When nutrient supply exceeds cellular requirements, adaptations to intracellular signalling occur, resulting in energy being stored as glycogen in muscle and the liver and fatty acids in adipose tissue. Overfuelling and aberrant fuelling of mitochondria result in oxidative stress, which not only disrupts cellular homeostasis but can alter epigenetic expression, with intergenerational effects. If the recycling mechanisms of the cell are insufficient to clear metabolic products, apoptosis may result or expression of Damage-Associated Molecular Patterns (DAMPs) on the cell surface may occur, activating immunity and inflammation at a systemic level. Disrupted cellular signalling affects cells with different “professional” functions in different organs, and it is the mechanism which underlies the associations between chronic non-communicable diseases such as cancer, type 2 diabetes, cardiovascular disease, neurodegenerative disease, autoimmune diseases, and macular degeneration. Mitochondria are the controllers of energy production and are pivotal in cell signalling. Mitochondrial function governs health at cellular and organismal levels. This paper reviews the influence of nutrition on mitochondrial function, nutrient sensing, autophagy, insulin signalling, and apoptosis—the key pathways in cellular homeostasis.

## 1. Introduction

Since about 1980, there has been an alarming rise in the global incidence of chronic non-communicable diseases affecting multiple systems of the body. This has coincided with the period following the introduction of National Nutritional Guidelines in the United States [1]. The associations between ultra-processed foods, obesity, insulin resistance, and chronic non-communicable diseases have been recognised, but the cell signalling pathways that connect them have not been widely integrated into care pathways in medical disciplines. As a result, the current medical paradigm is to focus predominantly on treating symptoms and disease rather than including the cellular causes and processes which lead to disease in disease management plans. In many cases, chronic non-communicable diseases can be effectively prevented and treated by modifying nutrition and lifestyle, thus reducing costly pharmacological and hospital interventions [2].

The biggest environmental changes that humans have experienced in the last half century are diet and exposure to environmental chemicals. The food we consume provides the fuel which mitochondria convert to energy, which in turn drives essential cellular processes. However, mitochondria do not simply generate energy, they also regulate cell signalling which aims to optimise cellular and organismal health in response to dietary input. The 3 million years of human evolution has resulted in a metabolism adapted to a diet which in geographical and environmental context included animal proteins and fats, as well as seasonally available plant foods, and to periods of relative nutrient scarcity [3]. The modern Western diet provides a nutritional surfeit as it contains an excess of refined carbohydrates, added sugar, and industrially produced polyunsaturated fats (PUFAs), which are detrimental to mitochondrial function. Many ultra-processed foods also contain added ingredients and agricultural chemicals which may have adverse health effects [4].

It is now accepted that mitochondria are endosymbiotic bacteria of the α-Proteobacteria phylum, which entered an Archaea host approximately 1.5 billion years ago [5]. With the maturation of the symbiosis, approximately 1500 of the original bacterial genes were transferred to the host nucleus, with the mitochondria retaining their own deoxyribonucleic acid (DNA) consisting of a ring of DNA containing 37 genes which encode for mitochondrial proteins and polypeptides required for oxidative phosphorylation [6]. The symbiosis between mitochondria and nuclear DNA is important: the nucleus contains approximately 1200 genes responsible for mitochondrial membranes, structure, and mitochondrial DNA repair [7].

Intracellular signalling and chemical interactions are complex and variable, depending not only on intracellular processes, but also responding to extracellular and environmental stimuli, and mitochondria are key regulators of these processes. This paper focusses on the key cell signalling pathways that are critical for health: redox signalling, insulin signalling and insulin resistance, nutrient sensing and autophagy, and apoptosis. For each of these, the “normal” pathways will be outlined and the influences of aberrant nutrition and environmental toxins and their relationship with chronic non-communicable diseases will be described.

## 2. Mitochondria, the Endoplasmic Reticulum, and the Integrated Stress Response

The basis of life in all organisms is the conversion of macronutrients in food into the energy which drives cellular processes. In human cells, by far the most important pathway for energy production is oxidative phosphorylation in the mitochondria (Krebs cycle) [8], which has numerous entry points for nutrients. The products, reduced nicotinamide adenine dinucleotide (NADH) and reduced flavin adenine dinucleotide (FADH2), provide electrons which enter the mitochondrial electron transfer chain [9], which resides in the inner mitochondrial membrane. This results in an electrochemical gradient which drives the production of the “energy currency” Adenosine Triphosphate (ATP) via ATP synthase (complex 5) [9].

Excess electrons react with oxygen and nitrogen to form reactive oxygen species and reactive nitrogen species (RONS) [10], which can act as signalling molecules [11]. When fuel intake and energy requirements are closely balanced, most of these are neutralised by mitochondrial and cytosolic antioxidants [12,13]. This produces other signalling molecules such as hydrogen peroxide (H_2_O_2_) and nitric oxide (NO) [11]. H_2_O_2_ is an important signalling molecule for processes such as hypoxic adaptation, apoptosis, regulation of phosphorylation signalling, cell growth and differentiation [9], and insulin signalling in neurons [14,15].

Relating cellular health to mitochondrial “function” or “dysfunction” is simplistic. Mitochondria in different organs differ morphologically and functionally, and they may recalibrate their features, activities, functions and behaviours in response to organismal demands and stressors. They have synthetic, signalling, and many other functions. They may transfer their DNA to other cells [16] and may themselves transfer intercellularly. They work with other organelles such as the endoplasmic reticulum (ER) and peroxisomes to adapt cellular activity to stimuli and stressors and are involved in regulating autophagy and apoptosis [17].

The endoplasmic reticulum (ER) occupies a large portion of the cytoplasm and is central to protein, lipid and carbohydrate synthesis, but it also regulates cellular function through membrane contact sites, which may be tripartite, with organelles including mitochondria, lysosomes and the Golgi apparatus. As the main intracellular calcium store, the ER transfers Ca^2+^ to mitochondria to maintain Krebs cycle homeostasis. Under pro-apoptotic conditions, excess Ca^2+^ enter the mitochondria, opening the permeability transition pores, disrupting the proton gradient and releasing cytochrome-c to trigger apoptosis. ER–mitochondrial contact sites also regulate mitochondrial fission and fusion in response to cellular metabolic state [18].

**Figure 2:**
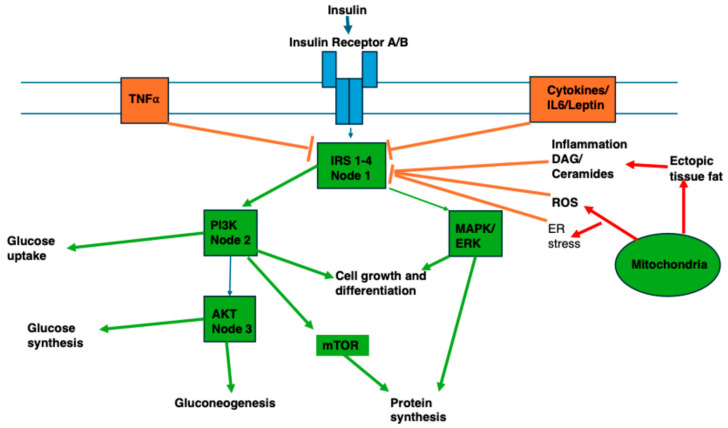
Simplified insulin signalling pathway. Orange: inhibitory to insulin signalling; Green: beneficial to cell health; Red: detrimental to cell and organismal health. IR A/B, Insulin receptor; TNFα, Tissue necrosis factor α; IL-6, Interleukin 6; IRS, Insulin receptor substrate; DAG, Diacyl glycerol; ER, Endoplasmic reticulum; MAPK, Mitogen-activated protein kinase; ERK, Extracellular signal-regulated kinase; PI3K, Phosphoinositide 3-kinase; AKT (also known as Protein kinase B).

The Integrated Stress Response (ISR) [19,20,21] is an intracellular signalling network that includes autophagy [22]. It allows the cell, tissue, and organism to maintain health by adapting to environmental and pathological variables such as impaired intracellular proteostasis (protein misfolding), nutrient deprivation, oxidative stress, and viral infection. An important form of cellular stress is ER stress, caused by such stimuli as hypoxia, oxidative stress, inflammatory cytokines, and nutrient deprivation, which results in protein misfolding. Misfolded proteins in the ER activate the unfolded protein response (UPR) [23], which results in insulin resistance in the liver, adipose tissue and skeletal muscle. This occurs as a result of reduced delivery of insulin receptors to the cell surface [24] and inhibition of the insulin signalling pathway (Figure 2). Mild ER stress is protective to pancreatic β cells, but severe ER stress leads to β cell death [25]. The UPR reduces protein synthesis by inhibiting mammalian target of rapamycin (mTOR) and altering messenger ribonucleic acid (mRNA) translation and the nuclear transcriptional program. If homeostasis is not restored, apoptosis is triggered. Misfolded proteins in the cytosol activate the heat shock protein (HSP) pathway [26], which also affects nuclear transcription to regulate protein synthesis. The ISR activates the transcription factor nuclear factor-κB (NF-κB) pro-inflammatory transcription factor and also stimulates the secretion of inflammatory cytokines such as IL-1β and IL-6. These inflammatory cytokines have downstream effects such as inhibiting insulin signalling. Different cell types have different optimal homeostatic set points for ISR activation or inhibition. The ISR acts in a rheostatic fashion rather than as an “on/off” switch [19].

## 3. Extracellular Vesicles

Homeostasis and stress responses rely on the ability of cells to regulate their own metabolic processes and to interact with their neighbours and remote organs. Cells produce signalling molecules, such as cytokines, which can perform this function and respond to extracellular chemical messages via receptors, for example, insulin and cytokines.

This raises the question of how these signalling molecules are transported to their destination and how the process is integrated with the cellular “machinery”. The answer lies in extracellular vesicles (EVs), lipid vesicles ranging from 40 nm to 1500 nm in size [27,28,29]. They are formed as part of the endosomal sorting complex required for transport (ESCRT). Vesicles are formed either by endocytic budding from the plasma membrane or by the Golgi apparatus. This generates “multivesicular bodies” (MVBs). MVBs can shuttle cargo to lysosomes for degradation or to the cell membrane for exocytosis as EVs. Cellular debris that exceeds the capacity of the autophagic process may be exocytosed in EVs.

EV cargo is dependent on the type and differentiation of the parent cell, as well as environmental influences. Cargo can include lipid mediators such as eicosanoids, proteins such as cytokines and growth factors, and genetic material such as mRNA and nuclear and mitochondrial DNA. Mitochondrial fragments or functional mitochondria may also be exocytosed in EVs. EV-mediated transfer of mitochondria is implicated in metabolic and immune regulation, as well as in the pathogenesis of cancer, liver disease, infectious disease, cardiovascular disease, and respiratory disease [30]. EVs may be activated by pathological stimuli such as inflammation, hypoxia, oxidative stress, cell death, and bacterial toxins, but they are also involved in physiological processes, including cell growth and differentiation, organogenesis, tissue repair and regeneration, antigen presentation and immune response, and ageing [27,28,29].

Targeting of receptor cells by EVs is hypothesised to relate to their size and surface molecules [27]. EVs circulate systemically, can cross biological barriers such as the blood–brain barrier, and can be detected in all body fluids [28]. EVs play an indispensable role in transmitting regulatory signals between neighbouring cells and distant organs.

## 4. Redox Signalling

**Figure 1:**
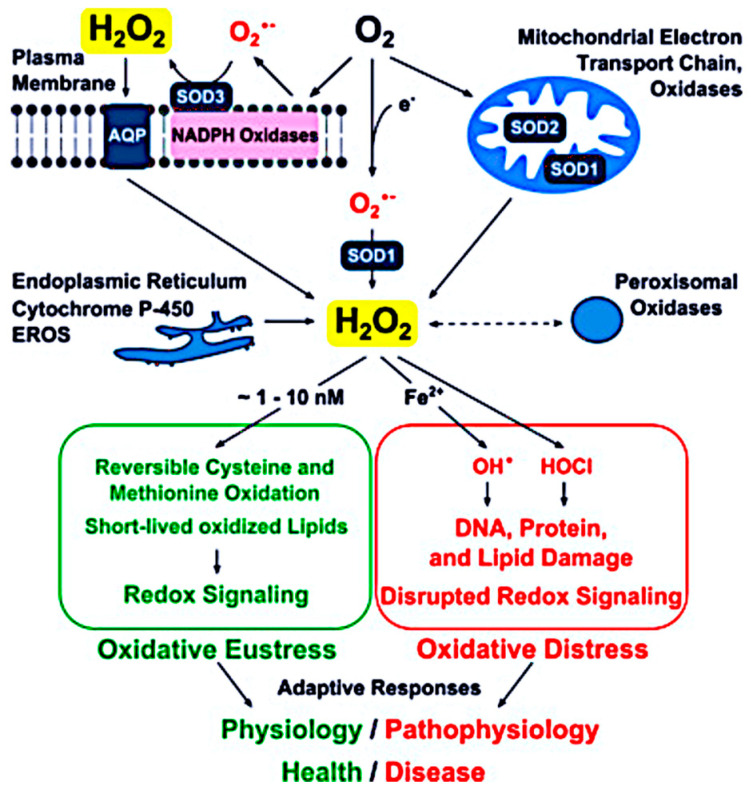
Redox signalling. AQP: aquaporin; SOD: superoxide dismutase; EROS: essential for reactive oxygen species. Helmut Sies. Hydrogen peroxide as a central redox signalling molecule in physiological oxidative stress: Oxidative eustress. *Redox Biology*, Volume 11, 2017, Pages 613–619, ISSN 2213-2317, https://doi.org/10.1016/j.redox.2016.12.035 [14].

It is widely accepted that “oxidative stress” is implicated in the aetiology of ageing and disease. However, the term “oxidative stress” refers to one half of oxidation–reduction (redox) reactions that are fundamental in cell biology [14,31,32,33]. Key redox mediators of essential cellular mechanisms are reactive oxygen, nitrogen and sulphur species, collectively known as reactive oxygen species (ROS). These are regulators of hormesis [34], the adaptive response to cellular and environmental (exposome) stressors [35]. ROS are maintained at equilibrium levels to facilitate physiological redox signalling. The normal level of ROS differs between cell types, being higher, for example, in progenitor cells. There is crosstalk between ROS species, and their effects are also dependent on duration of exposure [31]. Hydrogen peroxide (H_2_O_2_) acts as an important cell signalling molecule and is produced by redox pathways in mitochondria, the endoplasmic reticulum, peroxisomes, the nucleus, cytosol, and cell membranes (Figure 1). Concentrations of H_2_O_2_ are typically higher extracellularly than intracellularly. Lower concentrations are associated with proliferation, migration, angiogenesis, and adaptation to mild stress, along with Nrf-2 activation. This is known as redox eustress. Higher concentrations of H_2_O_2_ result in oxidative distress and are associated with NF-κB activation, inflammation, fibrogenesis, tumour growth and metastasis, and finally cell growth arrest and cell death [14].

A balanced redox state (redox eustress) in cells is physiological and is encouraged by balanced nutrition, intermittent fasting, and healthy lifestyle inputs such as exercise and sleep [36,37]. Redox eustress is mediated by nuclear factor erythroid 2-related factor (Nrf-2) [38,39], a transcription factor which is located in the cytoplasm in an inactive form. In response to oxidative stimuli, it is activated and translocates to the nucleus, where it binds to DNA, resulting in the production of antioxidants. Redox status is rebalanced, and cell functions, adaptations, resilience, and hormesis are preserved [32].

Where oxidant levels are low, for example, in hypoxia and ischaemia, a state of reductive distress prevails [40]. Reductive distress may occur with reduced mitochondrial activity, or when intracellular antioxidants exceed the requirements of the intracellular oxidative environment. There is evidence that antioxidant therapy may increase mortality [41] and prevent the health-promoting effects of exercise in humans [42]. Reductive distress activates another transcription factor, hypoxia inducible factor 1 (HIF-1α) [43], which acts as an oxygen sensor, to cause perinuclear accumulation of mitochondria and activation of a number of genes involved in glycolysis. The induction of increased ROS moves the cellular state towards redox eustress [32].

Oxidative distress is the situation that exists in many chronic non-communicable diseases. Poor-quality nutrition, a sedentary lifestyle, poor sleep, and environmental toxins are all implicated [4,32]. Excessive reactive oxygen species result in oxidative damage to proteins and DNA and lipid peroxidation, thus impairing cellular function. The cellular response to oxidative distress is mediated by NF-κB, which is also activated by cell surface ligands such as cytokines and (DAMPs). The effects include the production of antioxidants and are pro-inflammatory, although this is cell- and context-dependent [44,45].

## 5. Insulin Signalling and Insulin Resistance

Insulin is a 51-residue anabolic peptide hormone, the majority of which is synthesised in the beta cells of the Islets of Langerhans in the pancreas [46]. Pancreatic insulin secretion is stimulated primarily by glucose, which enters the beta cells via (non-insulin-dependent) GLUT 2 transporters. The glucose is “sensed” by glucokinase, which phosphorylates the glucose. Subsequently, glycolysis and mitochondrial respiration result in the production of ATP and the closure of ATP-sensitive potassium channels. This results in membrane depolarisation, calcium influx into the cell, and the fusion of insulin-containing vesicles with the cell membrane (exocytosis) [47]. Amino acids contained in dietary protein also cause secretion of insulin from beta cells, although to a far lesser extent than glucose [48]. While a major role of insulin is to regulate blood glucose, insulin is also essential for other cellular functions, which broadly speaking are anabolic and anti-apoptotic. Other cells such as neurons, glial cells [49], and retinal pigment epithelial cells [50] produce their own insulin and are unaffected by systemically circulating insulin.

All cells in humans express insulin receptors, which have A and B isoforms, on their cell membranes. In the brain, neuronal cells express the A isoform of the insulin receptor, whereas glial cells and cells in other tissues express both isoforms. The intracellular effects of insulin binding to the receptor are mediated by intracellular signalling pathways [51]. These pathways contain common upstream features in different cell types [52,53], but downstream effects differ [54], depending on their “professional” function [55], and are influenced by other factors such as nutrition, redox status and inflammatory mediators [56]. Figure 2 is a simplified representation of the insulin signalling pathway and insulin resistance (adapted from [52,57]).

Insulin regulates glucose uptake for energy metabolism via GLUT4 glucose transporters [58], which are present in skeletal muscle, the heart, adipose tissue and neural tissues, including the hippocampus, hypothalamus, cerebellum and retina. Although circulating insulin has limited passage across the blood–brain barrier, neurons can synthesise insulin locally, while cerebral glucose uptake is mainly mediated by insulin-independent GLUT1 and GLUT3 transporters [59]. In the brain, insulin signalling is crucial for non-metabolic functions such as neurodevelopment, neuroprotection, neurite growth, appetite regulation and fertility [15,60]. Unlike its anabolic peripheral effects, brain insulin has a net catabolic (anorexigenic) role. Neuronal insulin signalling requires a threshold burst of mitochondrial H_2_O_2_ for activation [15], whereas in skeletal muscle excess H_2_O_2_ inhibits insulin signalling [61].

Insulin resistance is a feature common to most chronic non-communicable diseases, including obesity, type 2 diabetes, cardiovascular disease, neurodegenerative disease, and cancer [54]. Circulating insulin levels are raised in insulin resistance, with some downregulation of cell membrane insulin receptors resulting from ER stress [24]. However, insulin resistance occurs mostly at the level of the intracellular pathway and is the result of cell membrane binding of inflammatory cytokines such as tumour necrosis factor α (TNFα) and IL-6, endoplasmic reticulum stress, fat metabolites such as ceramides and diacyl glycerol, and reactive oxygen species (Figure 2).

## 6. Nutrient Sensing

There are nutrient-sensing mechanisms for sugars, lipids, and amino acids [62]. Detailed exploration of these is outside the scope of this review, and interested readers are encouraged to access references [63,64].

For the purposes of this paper the balance between AMPK-activated protein kinase (AMPK) (catabolic) and mammalian target of rapamycin (mTOR) (anabolic), two key “hubs” of nutrient-sensing pathways will be discussed.

### 6.1. AMP-Activated Protein Kinase (AMPK)

Cellular homeostasis is dependent on the availability of energy and nutrients within a range that promotes normal cellular functions. Energy is produced predominantly by oxidative phosphorylation within the mitochondria, generating ATP. When cytosol proteins and enzymes are activated by phosphorylation, ATP is depleted, and adenosine diphosphate (ADP) and AMP levels are increased. If ATP production is insufficient for cellular needs, an increased ADP:ATP ratio activates AMPK, as does elevated cytosol Ca^2+^, and this is the “canonical” pathway. AMPK is also activated by numerous drugs and naturally occurring plant chemicals [64]. “Non-canonical” activation of AMPK occurs in response to reactive oxygen species (generated by the mitochondria) and genotoxic agents [64].

Activation of AMPK inhibits anabolic pathways (mTOR, see below) and promotes catabolic pathways. Cellular glucose uptake is enhanced by activation of GLUT1 transporters, which are present on most cells, although muscle and adipose rely more on insulin-dependent GLUT4 transporters. Fatty acid uptake into mitochondria is also enhanced. These pathways provide a substrate for energy production when nutrient intake is low. AMPK also initiates autophagy, the recycling process which provides other chemicals necessary for cellular homeostasis. AMPK is integral to mitochondrial fission, fusion, biogenesis, mitophagy, and motility, and together these processes match cellular energy requirements with nutrient availability [65,66].

At a systemic level, AMPK is also involved in regulation of appetite, responding to neuropeptide Y- and agouti-related protein-expressing neurons (NPY/AgRP neurons), pro-opiomelanocortin (POMC neurons), leptin, ghrelin, and adipokines in the hypothalamus [64]. Whole-body energy balance is influenced by AMPK, which promotes the release of catecholamines, and thus glucose mobilisation, in response to low glucose detected in the ventromedial hypothalamus [64].

AMPK also plays a role in circadian rhythm [67], regulation of the cell division cycle [68], and neural function [64]. In summary, AMPK is a crucial nutrient sensor whose function is to stabilise energy production and cellular substrate availability when nutrient intake is below cellular energy and nutrient requirements.

### 6.2. Mechanistic Target of Rapamycin (mTOR)

Broadly speaking, AMPK and mTOR [69] are two sides of an equation. AMPK is activated by nutrient scarcity and initiates pathways which provide energy and other essential cellular substrates by autophagy. Conversely, mTOR is activated by adequacy of energy, nutrients, and growth hormones and activates cell survival, growth and proliferation. AMPK stimulates autophagy and mTOR inhibits it [69].

mTOR forms two multiprotein complexes, mTORC1 and mTORC2, which are composed of discrete protein-binding partners to regulate cell growth, motility, and metabolism. These complexes are sensitive to distinct stimuli. mTORC1 is sensitive to nutrients, integrating signals from oxygen, growth factors (such as insulin), amino acids, and cellular energy to regulate cell growth by promoting lipid, nucleotide, and protein synthesis. It also enhances nuclear-encoded mitochondrial RNA production, leading to mitochondrial biogenesis and increased ATP generation [70]. Key amino acids activating mTORC1 include leucine, arginine, and glutamine, while hypoxia and cellular stress (e.g., excess reactive oxygen species) inhibit its activation [70].

mTORC2 is regulated via PI3K and is dependent on mTORC1 and other upstream influences, such as insulin and other growth factors, for activation (see Figure 2). The downstream effects of mTORC2 activation are cytoskeletal remodelling, cell migration, cell proliferation and cell survival.

mTORC1 controls the balance between anabolism and catabolism in response to fasting and feeding. While the “build” and “store” functions of mTORC1 are essential for cellular and organismal health, periods of inactivation of mTORC1 and activation of AMPK/autophagy are also essential to allow clearance of intracellular damaged proteins, lipids, and mitochondria. Imbalance of the mTOR and AMPK pathways in the mTOR direction is implicated in the chronic non-communicable diseases associated with the Western diet and lifestyle [69]. mTORC1 plays an important role in glucose homeostasis, affecting both pancreatic β cell and liver function. In the liver, the normal response to fasting is induction of autophagy (via AMPK), gluconeogenesis, and production of ketone bodies. Chronic mTORC1 activation stimulated by the overfeeding that is characteristic of Western dietary habits results in insulin resistance, pancreatic β cell failure, adipose deposition, and muscle atrophy—features of many chronic non-communicable diseases [69]. mTOR is also implicated as a player in immunity, cancer, brain function, and ageing [69]. Chronic mTOR activation may be a response to mitochondrially induced oxidative stress and exerts detrimental cellular effects by inhibiting mitophagy [71,72].

## 7. Autophagy

**Figure 3:**
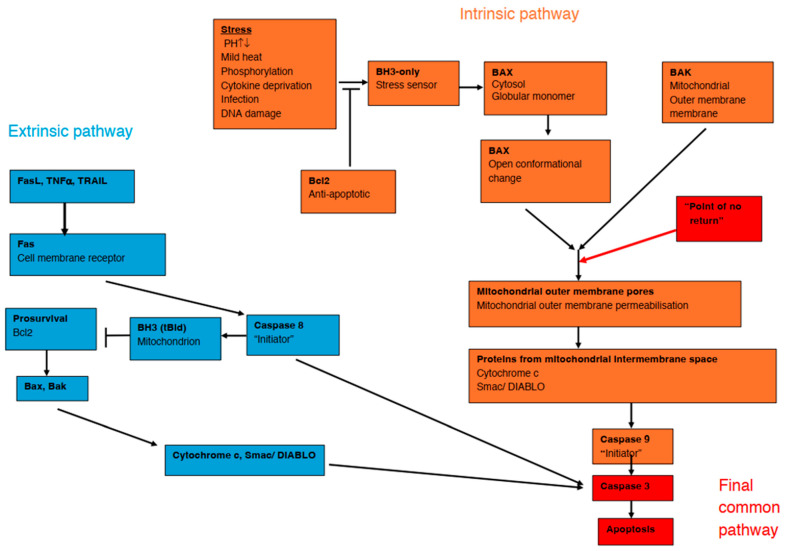
Intrinsic and extrinsic apoptosis pathways and the final common pathway. Fas (CD95 or Apo-1): Transmembrane protein receptor belonging to TNF superfamily that acts as a “death receptor”. Fas-L (CD95L or Apo-1L): Fas ligand, a transmembrane protein that triggers apoptosis when it binds to its receptor Fas. TRAIL (tumour necrosis factor-related apoptosis-inducing ligand): A cytokine that binds to death receptors, activating a caspase cascade that leads to apoptosis, and selectively targets transformed cells, crucial in immune surveillance by NK cells, T cells, and macrophages. Bcl-2 (B-cell lymphoma 2): A protein located on the outer mitochondrial membrane that prevents release of cytochrome-c into the cytoplasm and acts as a pro-survival, anti-apoptotic protein. Bax (BCL2-associated X protein): A pro-apoptotic protein that permeabilises the outer mitochondrial membrane. Bak (Bcl-2 homologous antagonist/killer): A pro-apoptotic protein that permeabilises the outer mitochondrial membrane. BH3, tBid: Proteins that sense cellular stress that, when activated, inhibit Bcl-2 and trigger mitochondrial outer membrane permeabilization. Smac or DIABLO (second mitochondria-derived activator of caspases): Facilitates apoptosis by release of caspases by blocking inhibitors of apoptosis.

Autophagy is well known for its cellular homeostatic role in removing damaged organelles and molecules and in recycling cellular components to satisfy nutritional requirements when intracellular ATP and nutrient levels fall. In general, autophagy is reparative and encourages cell survival, but in some instances autophagic cell death, apoptosis or necrosis may result [73,74]. In mammals, pure autophagic cell death is not thought to play an important role in developmental programmed cell death, but it may be involved in pathological cell death [74]. There is a complex interplay between autophagy and apoptosis which is mediated by the balance of stressors and pro-and anti-apoptotic proteins (such as BCL2 and BH3 only) (see Figure 3) [75]. Autophagy is part of the ISR [19,22] and responds to both endogenous and exogenous stimuli. Examples of endogenous stressors are amino acid or lipid starvation, reduced trophic factors or hormones, impaired intracellular cholesterol trafficking, ER stress, damaged protein products, redox stress, and mitochondrial damage. External stimuli include DAMPs, pathogen-associated molecular patterns (PAMPs), and TNFα [76].

There are three forms of autophagy: macroautophagy, microautophagy, and chaperone-mediated autophagy (CMA). Macroautophagy involves a large part of the cytoplasm and cell contents. The cargo is selective and determined by molecular signalling. A double-membraned autophagosome engulfs the cargo and fuses with a lysosome to form an autolysosome which enzymatically degrades the contents for recycling [74]. In microautophagy, lysosomes directly engulf small volumes of cytosolic substrate. CMA is stimulated by physiological stresses such as starvation. The target proteins bind to a heat shock protein and are delivered directly to a lysosome [73].

Autophagy is regulated by 16–20 evolutionarily conserved autophagy-related genes (ATGs) which encode the ATG proteins involved in autophagosome formation [77]. These autophagy proteins are also part of the autophagy pathway by which pathogens are targeted to the lysosome. The ATGs and the proteins they regulate mediate innate and immune responses to pathogens, which results in resistance to infection or may result in cell death [73,76]

## 8. Apoptosis

Apoptosis is an energy-requiring programmed cell death that maintains tissue homeostasis by enacting phagocytosis of cells that are damaged or redundant in a process that does not provoke inflammation [78]. It plays a crucial role in the regulation of embryological development and in maintaining optimal function in mature organs. Dysregulation of apoptosis is implicated in a variety of chronic non-communicable diseases, for example, cancer and autoimmune disease where apoptosis is suppressed, or neurodegeneration, where apoptosis is overactive [79]. Balance of apoptosis is dependent on signalling pathways which respond to intracellular and extracellular stimuli.

There are two apoptotic pathways, intrinsic and extrinsic (death receptor) (Figure 3). The intrinsic pathway is mediated by the mitochondria [80] in response to intracellular stressors such as alterations in pH, mild heat, phosphorylation, cytokine deprivation, infection, and DNA damage. The extrinsic pathway is activated by binding of extracellular ligands such as TNF-α, TRAIL (TNF-related apoptosis-inducing ligand [81]), and FasL (Fas ligand, a member of the TNF superfamily [82,83]) to the Fas cell membrane receptors (also known as CD95 or APO-1, a transmembrane protein belonging to the TNF receptor superfamily [82,84]).

Both pathways activate initiator caspases [85] and converge on a “point-of-no-return” final common pathway which activates the “effector” caspase 3 (Figure 3). Crosstalk between the intrinsic and extrinsic pathways exists. Interestingly, it seems that caspase 3 can play a non-apoptotic signalling role in neurodevelopment and neuroplasticity [86].

This brief overview of apoptosis in tissue homeostasis illustrates that under- or overactivation of apoptosis can be involved in disease causation. The two apoptotic pathways are regulated by signalling stimuli that are causally connected to the intracellular environment and circulating molecules. The mitochondria control the intracellular environment in response to fuel (food) quantity and quality and influence the extracellular environment by affecting transcription pathways that produce chemicals that in their turn react with cell membrane receptors and their signalling pathways.

## 9. Nutrition and Cell Signalling in Chronic Non-Communicable Diseases

### 9.1. Fructose

Increased fructose consumption is a component of increased consumption of ultra-processed foods, which also contain seed oils (see below), wheat flour, and food additives such as colourants, preservatives, and emulsifiers [87]. Globally, daily caloric availability [88] and intake per person have risen from 2352 kcals to 2828 kcals between 1961 and 2011 [89]. Changes in dietary behaviours have been accompanied by increasing sedentarism, which is also associated with the increase in chronic non-communicable diseases [90]. Fructose [91] is one of the sugars found in foods, most commonly in naturally occurring sources, such as fruits, where it may also be bound to glucose to form sucrose (“sugar”). In industrially produced high-fructose corn syrup (HFCS), glucose and fructose exist as monomers. Added sugar in the Western diet has increased from 2.9 kg/per person/per year in 1822 to 49 kg/per person/per year (equivalent to 32 teaspoons per day) in 1999 [87], predominantly contained in sugar-sweetened beverages and ultra-processed foods. Ancestrally, seasonally occurring high dietary fructose consumption of fruit in the autumn may have conferred a survival advantage by inducing insulin resistance and fat deposition in preparation for food scarcity in the winter [92]. In contrast, excessive added sugar in the modern Western diet is strongly associated with metabolic syndrome, non-alcoholic fatty liver disease, and cardiovascular disease, and there is good evidence to suggest that fructose plays a significant role [93].

Sucrose is a disaccharide which is broken down in the small intestine into glucose and fructose. Fructose is absorbed via GLUT5 pathways into the gut epithelial cells. Some is phosphorylated by ketohexokinase (KHK) and converted into glucose, lactate, glycerate, and other organic acids, which then pass through the portal circulation to the liver along with a proportion of the fructose. Very little fructose reaches the systemic circulation.

Metabolism of both glucose and fructose begins in the cytosol. Glucose is metabolised to pyruvate, which is transported into the mitochondria to participate in oxidative phosphorylation. The high added sugar consumption in the Western diet results in elevated fructose and glucose loads. Elevated intracellular glucose can activate the aldose reductase pathway, which has a low affinity for glucose, but when it is activated it converts glucose to fructose, adding to the fructose load. Fructose does not enter the mitochondria. In the liver, fructose phosphorylation for energy production, lipogenesis and glycogen synthesis consumes ATP and depletes intracellular phosphate. Reduced phosphate activates AMP deaminase, increasing purine breakdown and intracellular uric acid, while inhibiting AMPK and autophagy pathways [94]. Because humans lack functional uricase, this promotes intracellular uric acid accumulation and hyperuricaemia [95]. Elevated uric acid acts as a pro-oxidant, impairing mitochondrial function, increasing oxidative stress and inflammation, and contributing to hepatic steatosis, insulin resistance, endothelial dysfunction [96] and impaired cardiac energetics; clinically, hyperuricaemia is associated with gout, cardiovascular, metabolic and renal disease [97].

In the liver, high dietary fructose load leads to the production of advanced glycation end products (AGEs), ROS production, and activation of pro-inflammatory pathways via the transcription factor NFκB [45,98]. Experimental evidence in rodents has demonstrated that high-fructose feeding results in increased oxidative stress, increased mitochondrial DNA damage, and reduced mitochondrial biogenesis [99]. A study by Lustig et al. demonstrated that isocaloric fructose restriction improved metabolic function and non-alcoholic fatty liver disease in obese children [100].

Jones et al. demonstrated that elevated fructose exposure causes metabolic reprogramming and promotes inflammatory cytokine production by lipopolysaccharide (LPS)-stimulated monocytes [101]. Dysbiosis with leaky gut is a consequence of the Western diet. This can lead to exposure of immune cells to LPS [1], which can contribute to the systemic inflammatory milieu created by the Western diet and a sedentary lifestyle.

Western eating patterns have turned a metabolic pathway that in evolutionary terms carried a survival advantage into a disruptor of cell signalling homeostasis and a driver of chronic non-communicable diseases.

### 9.2. Seed Oils and Dietary Fats

The causative role of seed oils in chronic non-communicable diseases is controversial and, as is often the case, nuanced. Seed oils are commonly marketed as “vegetable oils” and contain Polyunsaturated Fatty Acids (PUFAs). They are industrially manufactured from seed sources such as canola, sunflower, safflower, corn, and soybean. The first seed oil to enter the human food chain was cottonseed oil in the 1860s. Consumption of seed oils in the USA prior to this was zero, rising to 80 g/per person/per day in 2010 [87]. Seed oils are contained in many ultra-processed foods and are used for cooking. Saturated fats (SFAs) from animal foods and dairy increased by only a small amount in the same period. The history is comprehensively covered by Knobbe [87]. Data show that in the US the rise in chronic non-communicable diseases is correlated with the increase in dietary seed oils, even as carbohydrate consumption falls [87]. The role of dietary SFAs in the causation of chronic non-communicable diseases is also nuanced. It seems that in isolation they have no adverse health effects but that in combination with high carbohydrate intake, as in the Western diet, they upregulate intracellular ceramide production [102], which in turn inhibits the insulin signalling pathway [102] at the levels of IRS1 and Akt (see Figure 2). PUFAs are not building blocks for ceramide production.

Seed oils comprise omega-6 PUFAs, predominantly linoleic acid (LA), and omega-3 PUFAs, predominantly alpha linolenic acid (ALA). Omega-6 and omega-3 fatty acids cannot be synthesised by humans, and dietary consumption is “essential”. Historically, the dietary ratio of LA:ALA was 3:1 or less. In the modern diet, the ratio is typically greater than 15:1 [103]. This is associated with a 136% increase in adipose tissue LA in the US over the past half century [104]. LA and ALA share a competitive metabolic pathway, with conversion of ALA into the bioactive omega-3 PUFAs docosahexaenoic acid (DHA) and eicosapentaenoic acid (EPA) being inefficient. This results in a bias towards the pro-inflammatory products of LA metabolism, such as arachidonic acid (AA), versus the anti-inflammatory products of ALA metabolism. This has been implicated in chronic non-communicable diseases [103,105]. However, the production of AA from LA saturates above 2% (approx. 4–6 g) of caloric intake, and studies have shown that higher LA intake in humans does not elevate inflammatory markers [106]. Nevertheless, since LA can undergo β-oxidation in the mitochondria, excessive dietary LA intake may contribute to overfuelling and raised oxidative stress [6]. The effects of excessive dietary LA intake in humans are not clear and likely relate to dietary context, genetics, and oxidative state [61].

While the signalling properties of PUFA metabolites in inflammatory pathways are important, PUFAs are essential constituents of cell membranes [105,107]. They maintain membrane fluidity; act as signalling molecules which regulate inflammation and immunity [105]; regulate blood vessel calibre; and influence platelet aggregation, synaptic plasticity, cellular growth, pain, and sleep [103]. Membranes in different locations contain different amounts of PUFAs. The inner mitochondrial membrane has a protein-to-lipid ratio of 4:1. Regarding the lipid content, up to 20% is cardiolipin, 80–90% of which is linoleic acid (LA). The LA provides optimal structural conformation for the electron transfer chain proteins which are embedded in the inner mitochondrial membrane (IMM).

Inflammation alone does not explain the link between increased dietary PUFAs and insulin resistance, and human studies show no consistent association [108]. Both omega-3 and omega-6 PUFAs contain double bonds that make them susceptible to lipid peroxidation, producing metabolites such as 4-hydroxy-2-nonenal (4-HNE), which in animal studies induces insulin resistance by inhibiting insulin signalling via the IRS and Akt pathways [109] (see Figure 2). Although thermal food processing can generate oxidised linoleic acid metabolites (OXLAMs) [110], experimental evidence suggests that increased dietary linoleic acid raises tissue OXLAM levels primarily through endogenous peroxidation rather than from preformed oxidised lipids in the diet [111].

Mitochondrial inner membrane peroxidation and conformational change can occur secondarily to mitochondrial oxidative stress induced by over- and inappropriate fuelling [6]. Intracellular lipid peroxidation underlies the activation of the pro-inflammatory signalling cascade (NF-κB), ER stress, impaired insulin signalling, activation of AMPK [112], and potentially apoptosis or necrosis [113].

## 10. Transcription Factors, Epigenetic Reprogramming, and Environmental Toxins

Cellular responses to oxidative stress and inflammation aim to maintain homeostasis. Stressors activate the ISR, which influences mitochondrial gene transcription [114,115], as well as the anti-oxidative transcription factor Nrf-2 [116] and the pro-inflammatory transcription factor NF-κB [116], which govern alterations in the nuclear epigenome [117]. There is a crosstalk between mitochondrial and nuclear DNA [118], and the mitochondria communicate with the nucleus via numerous chemical messengers [119]. Homeostasis is achieved when mitochondrial function is balanced with the oxidative state of the cell [118].

The modern environment exposes humans to a multitude of toxic chemicals [4]. Work by Heindel et al. has demonstrated that these chemicals are implicated in obesity and chronic non-communicable diseases by binding to cell and hormone receptor sites and modifying the epigenome. Worryingly, these epigenetic effects may occur prenatally and can be intergenerationally transmitted [120,121,122].

## 11. Conclusions

When underlying cellular metabolic processes are considered, their associations with various chronic non-communicable diseases are understandable. Human health is dependent on the integrated responses of the organs to nutrition and the environment. These responses have evolved over at least 3 million years and are genetically determined, providing homeostatic mechanisms which adapt survival and thriving to conditions, such as food scarcity and plenty, and climate [92,95]. At a cellular level, the responses are governed by the mitochondria, which provide not only energy but also a range of signalling cues in the form of reactive oxygen species and other molecules. When we stay within this range, all is well. Currently, we live outside this range. Our dietary behaviour and environment are not matched to our evolutionary programming. That is why we witness the present-day epidemic of chronic non-communicable diseases.

For the past half century, our understanding of diseases has progressed and lifespan has lengthened, although healthspan has not benefited as much as we may have hoped. Many people spend the last decade of their lives in declining health, with loss of mobility and independence, and this is largely due to the effects of chronic non-communicable diseases. Over 70% of US adults are overweight and 35% are obese, with chronic non-communicable diseases consuming the largest part of the health budget [1,123].

The purpose of this paper has been to look “behind” the metabolic markers that signify chronic non-communicable diseases, to keep asking “why” and to follow the “backward” trail through the complex cell signalling cascades. Followed far enough, all paths lead to the mitochondria. Without mitochondria there would not be sufficient energy to support cellular processes and life. For optimum cellular health, mitochondria require the right fuels in the right amounts. Healthy cells create healthy organs and a healthy human, with intracellular and hormonal signalling maintaining homeostasis. Variations in nutritional and environmental conditions result in adaptations in cell signalling, maintaining homeostasis, and this is healthy. When these conditions are outside the “homeostatic range” for too long, there is a risk of adaptations that can damage health. Examples are overnutrition, poor food quality (ultra-processed foods), starvation, a sedentary lifestyle, poor sleep, social isolation and chemical exposure.

Of course, healthcare practitioners should prioritise treatment of disease and mitigation of suffering, but we need to “see things differently” [124] and treat the causes and processes underlying disease, rather than focussing on symptoms and markers of disease. Moving the medical paradigm “upstream” can be achieved by raising awareness of the food, lifestyle, and environmental effects on cell signalling and health in pre- and postgraduate medical education. Unwin et al. [125] have demonstrated that remission of type 2 diabetes can be achieved by diet and lifestyle intervention. Numerous models exist for modifying health by non-pharmaceutical means, but there is no consensus on which is best. Assisting patients with habit change is fundamental in any model. Research to establish an effective and cost-effective model which can be incorporated into public health systems should be prioritised. Since the treatment of non-communicable diseases consumes approximately 85% of the US health budget and they are responsible for 87% of deaths, the potential economic benefits are substantial. The potential to improve health is beyond question.

## Data Availability

No new data were created or analysed in this study.
